# A New Rubber Clamp

**Published:** 1883-06

**Authors:** 


### A New Rubber Clamp.

We have received and used for a short time, what may properly be called, a double hinged rubber clamp. This instrument adjusts itself to a tooth of any form quite accurately, much better than any clamp that has been presented hitherto.

About three sizes of this form of clamp would, as it now seems, be sufficient for all cases, meeting each case better than can be done with a large number of the ordinary clamps. It is the device of Dr. J. P. Carmichael, of Milwaukee. It will within a short time be in the market, when all will have an opportunity to try it.

